# Understanding the Effects of Binders in Gas Sorption and Acidity of Aluminium Fumarate Extrudates

**DOI:** 10.1002/chem.202103420

**Published:** 2021-12-10

**Authors:** Miguel Rivera‐Torrente, Danny Kroon, Marie‐Vanessa Coulet, Carlos Marquez, Nikolaos Nikolopoulos, Rifan Hardian, Sandrine Bourrelly, Dirk De Vos, Gareth T. Whiting, Bert M. Weckhuysen

**Affiliations:** ^1^ Inorganic Chemistry and Catalysis, Debye Institute for Nanomaterials Science Utrecht University Universiteitsweg 99 3584 CG Utrecht The Netherlands; ^2^ MADIREL (UMR 7246) Aix-Marseille University, CNRS Centre de St Jerôme 13013 Marseille Cedex France; ^3^ Centre for Membrane Separations, Adsorption, Catalysis and Spectroscopy for Sustainable Solutions (cMACS) KU Leuven Celestijnenlaan 200F Box 2454 3001 Leuven Belgium

**Keywords:** acidity, aluminium fumarate, CO_2_/CH_4_, metal-organic frameworks (MOFs), solids extrusion

## Abstract

Understanding the impact of shaping processes on solid adsorbents is critical for the implementation of MOFs in industrial separation processes or as catalytic materials. Production of MOF‐containing shaped particles is typically associated with loss of porosity and modification of acid sites, two phenomena that affect their performance. Herein, we report a detailed study on how extrusion affects the crystallinity, porosity, and acidity of the aluminium fumarate MOF with clays or SiO_2_ gel binders. Thorough characterization showed that the clay binders confer the extrudates a good mechanical robustness at the expense of porosity, while silica gel shows an opposite trend. The CO_2_ selectivity towards CH_4_, of interest for natural gas separation processes, is maintained upon the extrusion process. Moreover, probe FTIR spectroscopy revealed no major changes in the types of acid sites. This study highlights that these abundant and inexpensive clay materials may be used for scaling MOFs as active adsorbents.

## Graphical abstract

**A pinch of binder, a pinch of MOF**: The effect of co‐extruding aluminium fumarate with either clays or silica gel binders has been investigated. Textural, microscopic, and spectroscopic techniques show the changes in porosity and surface sites, and CO_2_ and CH_4_ adsorption isotherms fitted an ideal Jensen‐Seaton adsorption model. We have demonstrated the viability of extrusion for MOF extrudate formation and the changes associated to the process.

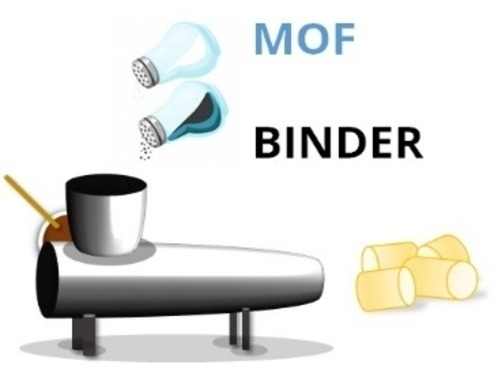

## Introduction

Metal‐Organic Frameworks (MOFs) are a class of porous coordination polymers that have been intensely investigated for their applications in a broad range of fields, such as catalysis, chemical sensing, drug delivery or gas sorption and separation.[^1^, ^2^] Although their potential has been proven in many proof‐of‐concept studies, the majority of them were performed with powdered samples, hampering their direct implementation as functional materials at the industrial scale. This requires further understanding of their shaping process, as this can have an impact on their properties and thus on their performances.^[3]^ Very often, MOFs suffer from a strong decrease in available surface area, pore volume or crystallinity upon compaction into tablets or pellets.[^4^, ^5^] Recent studies concerning sol‐gel synthesis methods have offered an alternative to traditional shaping technologies,[^6^, ^7^] but applicability of this strategy to a wide range of MOFs has not yet been proven. Different classical techniques have been reported for the shaping of MOFs, such as granulation,[^8^, ^9^] pelletization,[^5^, ^10^, ^11^, ^12^, ^13^] coating (e. g. spray‐drying, washcoat or spin‐coating)^[14]^ or even foaming production.[^15^, ^16^] Another method is extrusion, in which the powdered material is mixed and compacted with another, so called, binder (e. g. clays, alumina, silica or organic polymers), that enhances the mechanical properties of the mixture during and after the extrusion process.[^17^, ^18^] A great example of extrusion with MIL‐53(Al) has been reported by Kriesten et al.,^[19]^ showing high gas sorption properties albeit with low mechanical strength for the typical requirements for industrial application.

**Figure 1:**
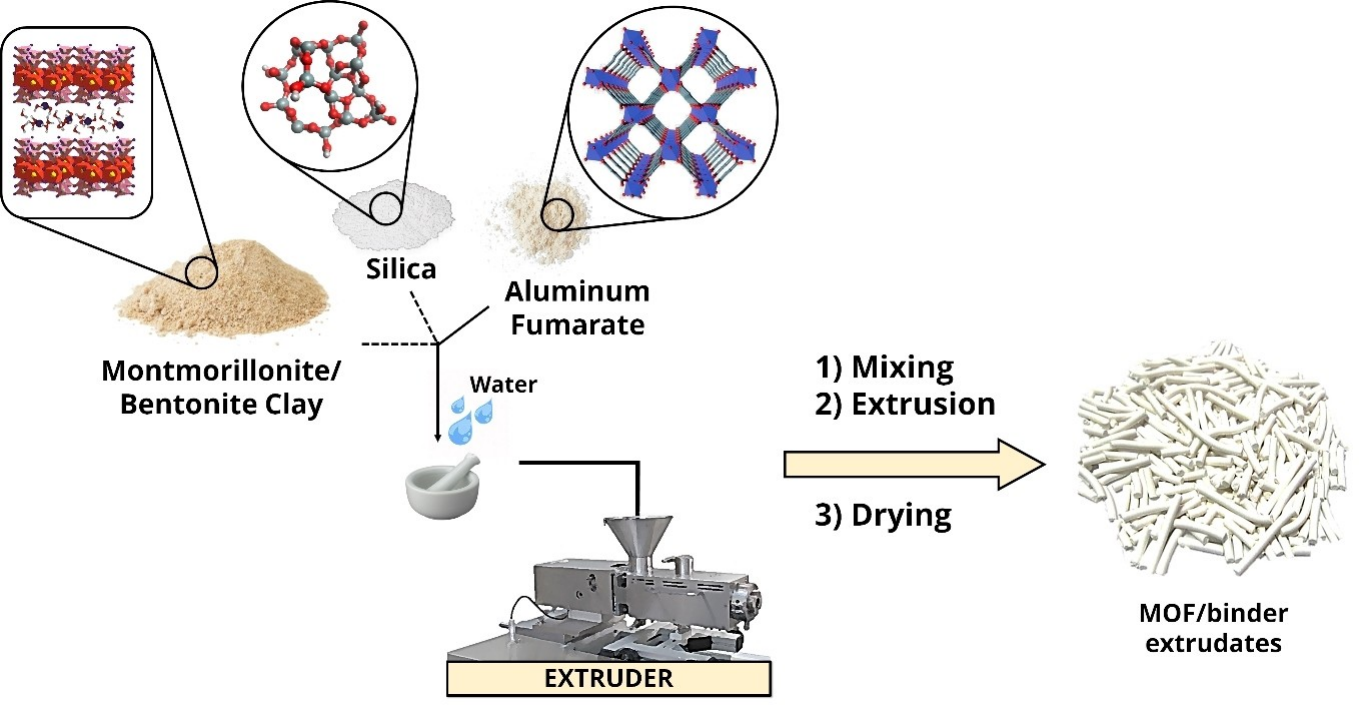
Preparation of the shaped bodies by extrusion of the water mixed paste consisting of aluminum fumarate MOF and binder (either montmorillonite, bentonite, or silica) and drying.

Due to its hydrothermal stability and relatively low cost, aluminum fumarate, [AlO_4_(OH)_2_(C_4_O_4_)],^[20]^ has been studied for different applications such as the separation of branched/linear hydrocarbons in the petrochemical industry[^21^, ^22^] or for water‐adsorbing heat pumps for efficient energy transformation.[^23^, ^24^, ^25^] Other proposed applications concern the adsorption of different gases (CO_2_ or CH_4_) for carbon capture, natural gas storage in the automotive industry or landfill gas separation.[^26^, ^27^, ^28^] Pellets with other binders such as metal–organic polyhedra,^[29]^ or even inorganic solids and clays such as the ones used in our study, have been used in the adsorption of these gases.^[30]^ Aluminum fumarate's performances are governed by the physicochemical and reactivity properties (e. g., acidity, porosity, and surface chemistry), in which OH bridging groups, that form electrostatic or dipolar interactions with adsorbate molecules, play a crucial role. It has been shown that the extrusion process, as well the binder choice, can alter the textural and catalytic properties of microporous materials, such as zeolites, whose properties strongly rely on the defects present in their molecular structure.[^31^, ^32^, ^33^, ^34^, ^35^] Thus, we sought to understand how the extrusion process performed with three different inorganic binders (montmorillonite and bentonite clays and a commercial silica) may affect the properties of aluminum fumarate in both catalysis and gas sorption. In this work, millimeter‐sized bodies were synthesized (Figure 1) and characterized using a broad array of techniques to study their structural stability (XRD), their textural characteristics such as morphology (FIB‐SEM‐EDX), porosity (N_2_ adsorption at 77 K and Hg porosimetry) and density (He pycnometry), their gas separation capacities toward CO_2_ and CH_4_ and their acidity (CO, py, and CD_3_CN‐probe FTIR spectroscopy).

## Results and Discussion

### Preparation of the Extrudate Bodies

First, characterization of the crystalline structure, porosity and thermal stability of the commercial aluminum fumarate was carried out by standard routine techniques (see Figures S1–S5 for XRD, N_2_ physisorption and TGA), showing that the materials properties are similar to those published in literature.^[36]^ The commercial aluminum fumarate shows a comparable BET surface area (S_BET_) to classical lab‐made materials (1034 vs. 1080 m^2^ ⋅ g^−1^). Interestingly, the sorption isotherm exhibits a hysteresis between the adsorption/desorption branches suggesting a type IV isotherm deviating from a purely type I isotherm, characteristic of microporous material. Barrett‐Joyner‐Halenda (BJH) method was used to evaluate the pore size distribution. It is worth mentioning that we focused on mesopores in this work, so the use of non‐local density functional theory (NLDFT) for calculating the micropore size distribution was not considered. A large fraction of mesopores of ca. 10 nm in size were evidenced (Figure S2), along a higher pore volume are also associated to it, both of which are probably related to its production process.

As explained in the experimental section, shaped bodies of inorganic materials formed by extrusion are typically prepared by including small amounts of water or other solvents, as well as plasticizers in order to reduce the shear and thermomechanical stress and their effects on the crystalline structure of the components (Figure 1).[^37^, ^38^, ^39^, ^40^, ^41^] In the case of montmorillonite and bentonite clays, the addition of small amounts of water (5–10 mL) resulted in robust cylindrical bodies post extrusion and calcination. In the case of the silica binder gel, addition of methylcellulose and propanolamine plasticizers^[42]^ was mandatory to obtain stable bodies. Once synthesized, the shaped bodies (both MOF/clay and MOF/SiO_2_ composites) were dried in air at room temperature overnight prior to calcination in a tubular oven under an air flow of 100 mL min^−1^ (and 5 K min^−1^ ramp) at 573 K for 5 h. Thermal stability of the composites throughout the calcination was verified by means of TGA (see Figure S5), which shows that the onset of MOF decomposition starts at ∼750 K, i. e. about 200 K higher than the calcination temperature utilized. In the case of MOF/SiO_2_ extrudates, calcination for 5 h resulted into dark brown color arising from degraded methylcellulose. Thus, a longer calcination program of 18 h had to be applied to further remove those impurities.

**Figure 2:**
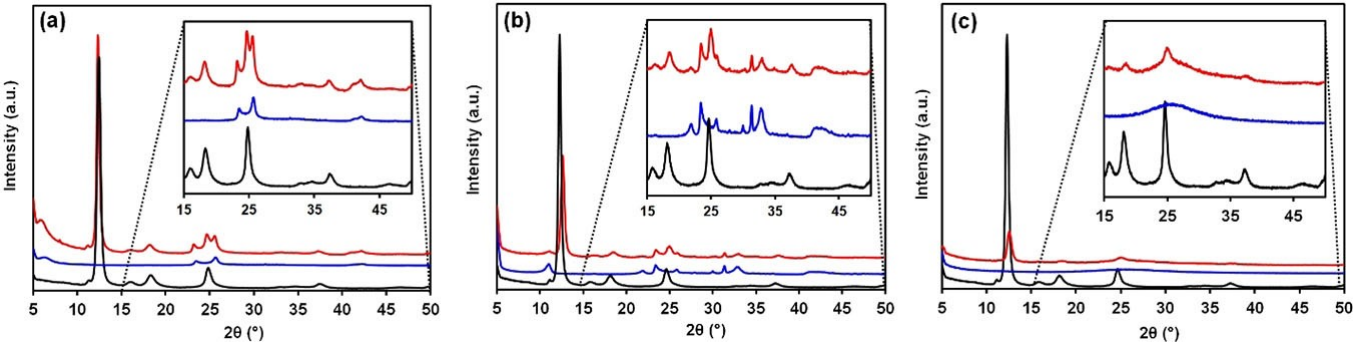
X‐ray diffraction (XRD) patterns showing the pure aluminum fumarate (black), the pure binder (blue) and the extrudate bodies (red); of (a) MOF/Mont, (b) MOF/Bent and (c) MOF/SiO_2_ shaped composites.

After preparation, the stability of the crystal structure of aluminum fumarate, as well as the binders, was studied by XRD (see Figure 2). The diffraction pattern of the MOF/Mont bodies shows the peaks corresponding to both components with similar intensities to those of the starting materials, indicating only a slight loss of crystallinity for MOF/Mont. However, the strong increase of the scattering at low 2θ values could be attributed to a partial amorphization. Extrusion and drying of the MOF/Bent bodies lead to strong decrease of the intensity of the main peak at 12.3°, indicating partial loss in crystallinity. A similar trend can be observed for the other reflections between 15 and 50°. At last, when SiO_2_ and methylcellulose were employed for the extrusion process, a sharp decrease in the peak was observed, as well as for the main MOF reflections, again indicating partial collapse of the crystallinity. Longer calcination times resulted in further loss of the crystal structure although the sign of a Bragg peak remains at 12.3° (Figure S6). It is worth noting a slight shift in the position of the peak towards higher 2*θ* values in the MOF/Bent and MOF/SiO_2_ patterns, which could mean a reduction in the cell parameter due to the compression to form extrudates, but further investigations are required to clarify if that is the case.

Another crucial feature of these composites that directly acts on their performances is porosity, which can drastically influence gas and liquid diffusion in adsorption and catalytic processes. To verify if the partial losses in crystallinity led to lower microporosity, which arises mainly from the MOF component, N_2_ physisorption isotherms at 77 K of the extrudates were compared to the pure aluminum fumarate powder (Figures S2 and S3). Table 1 shows how the mixing of the clay binders with the MOF powder resulted in values somewhat lower than the expected porosity of a 50 : 50 % wt mixture.

**Table 1:** Textural and mechanical properties of the extrudates obtained from Hg porosimetry and uniaxial compression tests.

Extrudate composition	Total intrusion volume [cm^3^ ⋅ g^−1^]^[a]^	Total pore area [m^2^ ⋅ g^−1^]^[a]^	Mesopores size [nm]^[a]^	Bulk porosity [%]^[b]^	Crush strength [N]^[c]^	Density [g^3^ ⋅ cm^−1^]^[d]^	BET surface area [m^2^ ⋅ g^−1^]^[e]^	C value (x=0)	V_micro_ [cm^3^ ⋅ g^−1^]^[e,f]^	V_meso_ [cm^3^ ⋅ g^−1^]^[e,f]^
MOF/Mont	0.39	152	∼20	39	62.6 (4.2)	1.97 (0.16)	595 (627)	1776	0.26 (0.20)	0.20 (0.21)
MOF/Bent	0.34	146	∼10	36	97.5 (18.1)	1.93 (0.35)	410 (517)	1200	0.23 (0.15)	0.13 (0.18)
MOF/SiO_2_	1.10	206	∼10/∼10^3^	62	–	2.01 (0.28)	397 (660)	626	0.67 (0.15)	0.24 (0.75)
MOF/SiO_2_^[g]^	1.40	250	∼10/∼10^3^	68	8.67 (2.1)	–	274 (660)	192	0.03 (0.15)	0.91 (0.75)
Mont	0.29	66	∼10/50	37	42.1 (0.8)	2.37 (0.06)	220	813	0.10	0.07
Bent^[h]^	0.13	38	∼20	24	57.0 (5.8)	2.96 (0.49)	–	–	–	–
MOF powder	–	–	–	–	–	–	1034	2149	0.28	0.46

[a] Obtained from the Hg porosimetry intrusion/extrusion experiments at 298 K. [b] Corresponds to the fractional pore volume of the bulk pellets ($\varphi =\frac{V-V_{s}}{V}=\frac{V_{p}}{V}\times 100$ ). Determined by uniaxial compression rupture tests (*σ_std_* of 5 measurements in brackets, compared to the rupture value of a reference commercial 4 Å molecular sieve, 40.9 (3.4)). [d] Values between parentheses show the *σ_std_* of 10 different He pycnometry measurements. [e] Numbers between brackets indicate theoretical values based on a 1 : 1 %wt mixture. [f] Calculated from the *t‐plot* method. [g] After 18 h of calcination in air flow. [h] Showed no adsorption of N_2_ at 77 K after drying at 623 K for 12 h in nitrogen flow.

**Figure 3:**
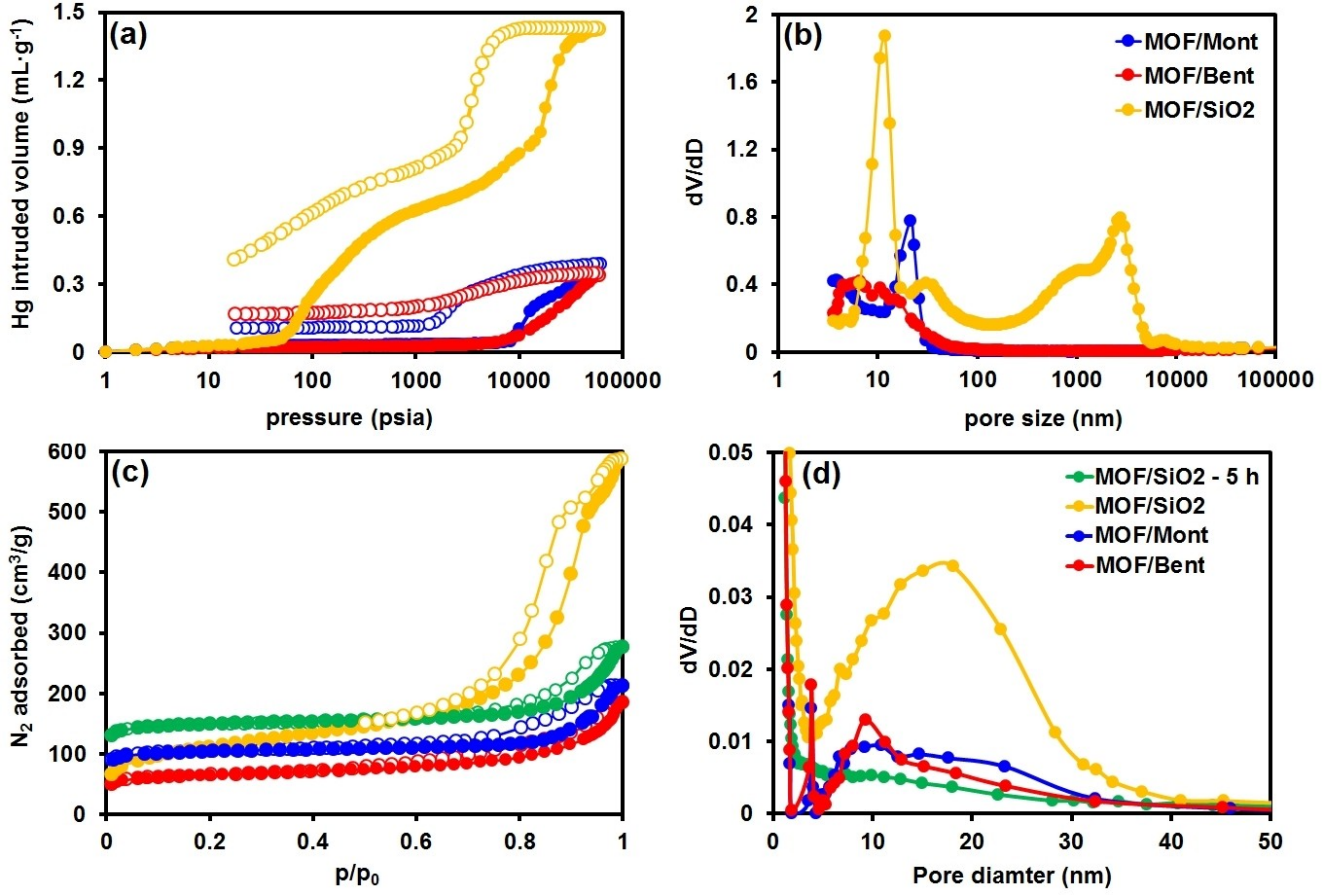
Hg porosimetry (a) intrusion and extrusion (empty circles represent the Hg extrusion cycle), (b) pore size distribution (PSD) curves, (c) N_2_ adsorption isotherms at 77 K; and (d) Barrett‐Joyner‐Halenda (BJH) PSDs from the desorption data of the (blue) MOF/Mont, (red) MOF/Bent and (yellow) MOF/SiO_2_ after 18 h and (green) MOF/SiO_2_ after 5 h of calcination extrudates.

This may be ascribed to either diffusion of charge‐compensating cations (i. e., Ca^2+^, Mg^2+^ and Al^3+^) into the aluminum fumarate pores, or direct pore blockage of the 1D channels by physically deposited binder on the MOF surfaces. When silica gel was utilized as a binder (Figure 3c), a much lower fraction of mesopores is observed after a calcination period of 5 h, despite the presence of those in the pure SiO_2_ binder material (Figure S8). However, after 18 h of thermal treatment, the isotherms and pore size distribution, show an enhancement of the available surface area as well as the presence of ca. 20 nm mesopores. Hence, the removal of the plasticizers requires harsher calcination conditions to remove the impurities present in the extrudates, resulting in the associated partial collapse of the pore structure. It is worth noting that extrusion of pure SiO_2_ resulted in brittle extrudates with poor mechanical stability.

A complementary characterization of the textural properties was carried out by Hg intrusion‐extrusion experiments. Such measurement aimed at determining if the formation of other types of pores occurred during extrudate preparation. Indeed, non‐wetting mercury is unable to penetrate the micropores of the MOF material, probing exclusively meso‐ and macropores generated during the extrusion process. The intrusion/extrusion cycles of the MOF/Mont and MOF/Bent extrudates are presented in Figure 3a together with the pore size distribution (Figure 3b). The values, summarized in Table 1, are in good agreement with nitrogen sorption data since they indicate the presence of mesopores in the 10 to 50 nm range. Interestingly, the extrudates show similar degrees of bulk porosity (36 and 39 %, for montmorillonite and bentonite, respectively), which are comparable to the extrudates containing exclusively clay material (Figure S7). This suggests that the fraction of available pores in the mesopore size range is dominated by the mesopores formed between the binder (Table 1) and the MOF, and those of the MOF component of the extrudate as no mesopores are present in the montmorillonite and bentonite clays. Again, a difference can be seen when silica gel is used as a binder material. In this case, in addition to the mesopores in the 20 nm range (Figure S8) larger pores of 0.4–3 μm are evidenced in the extrudates (Figure 3b). A porosity value nearly twice as high as that of the MOF/clay extrudates is obtained, as well as a higher total volume of Hg infiltrated, i. e., 1.1 vs. 0.35–0.4 cm^3^ ⋅ g^−1^. Thus, it is evident that although crystallinity of the MOF is not fully preserved during extrusion, the material remains highly porous. Once more, calcination for longer periods of time for MOF/SiO_2_ (Table 1) resulted in a slight enhancement of the porosity, with 62 % after 5 h, and 68 % after 18 h of calcination in air flow. Additionally, the BJH distribution confirmed the presence of mesopores of ca. 15 nm in diameter, arising from the SiO_2_ binder.

**Figure 4:**
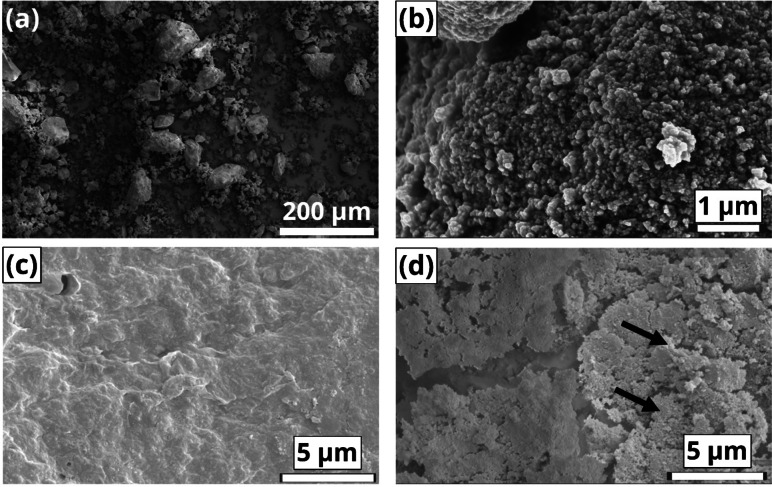
SEM micrographs of (a,b) aluminum fumarate at different magnification levels. Images of the side‐view (c) and (d) cross‐section after FIB‐sectioning of a MOF/Bent extrudate. Arrows in (d) highlight the MOF and extrudate grains compacted together.

Besides crystallinity and porosity, other key properties of shaped bodies are their mechanical robustness and compaction of the components. Crush strength tests were carried out (Table 1), by compressing the pellets and measuring the crushing strength. The MOF/Mont and MOF/Bent extrudates showed high mechanical stability, with values largely surpassing the commercial 4 Å molecular sieve (40.9 N; ∼100 N/mm^2^), and even those of the extrudates purely consisting of clay. This indicates that the MOF provides mechanical strength rather than fragility to the extrudates, probably due to an effective packing of the nanosized MOF crystallites with the clay.^[43]^ In contrast, the highly porous MOF/SiO_2_ bodies showed poor mechanical stability, which may limit their applicability, i. e. attrition resistance, together with the lesser strength of SiO_2_ compared to the crystalline clays. To study the intimacy of the components upon mixing, both side‐views and cross sections images of the extrudates were acquired by means of SEM. On one hand, Figure 4a and 4b show the pristine aluminum fumarate, which consists of ∼ 100 nm crystals, probably arising from a fast crystallization, aggregated in larger micron‐sized particles. Figure 4c,d show micrographs of the MOF/Bent side‐view, where a smooth, fully densified surface can be observed. The dense packing of aluminum fumarate and binder are in line with the crushing tests, in which these extrudates showed the highest mechanical strength. Imaging of the cross‐section after applying focused ion beam (FIB)‐sectioning revealed the presence of some MOF aggregates in close contact with the binder.

**Figure 5:**
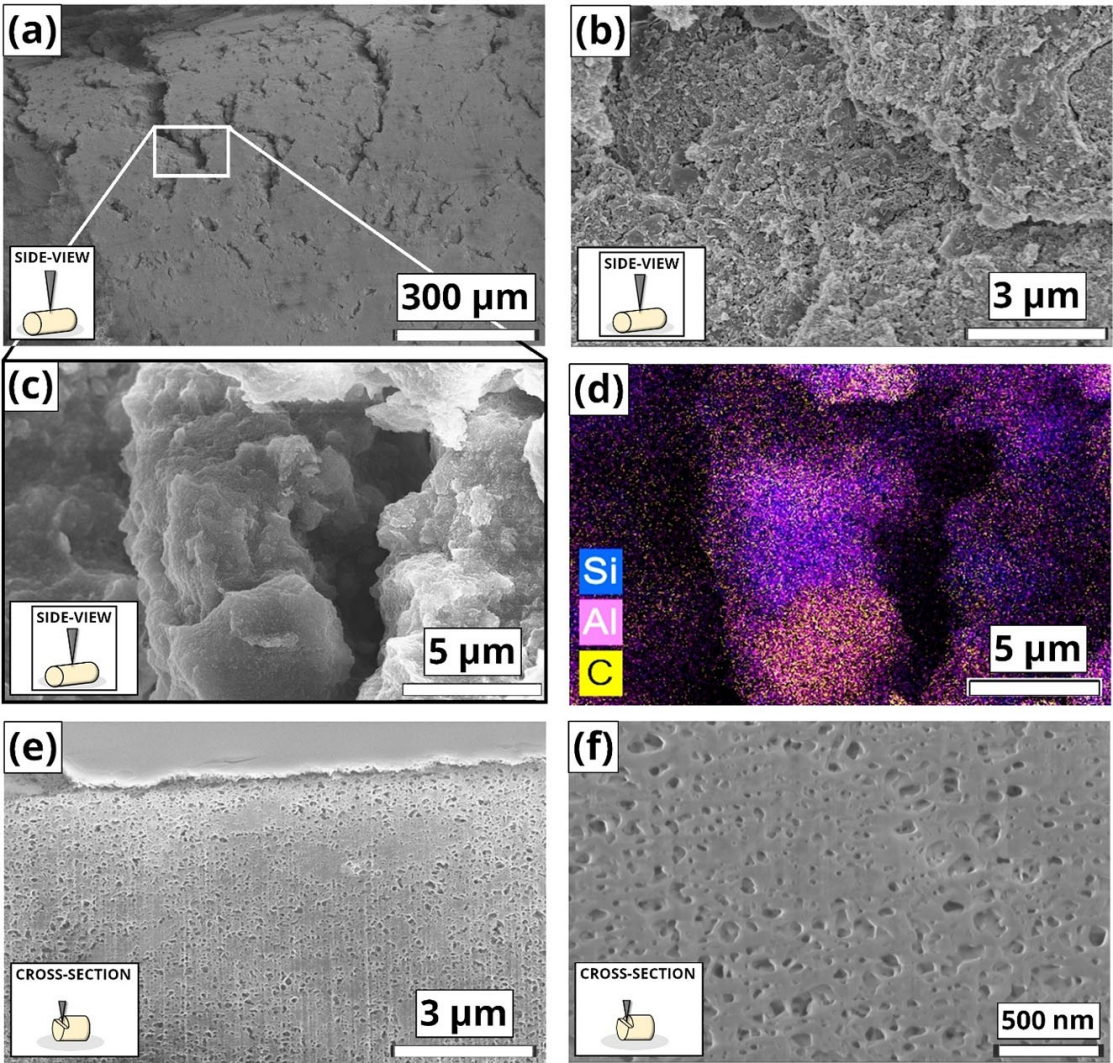
SEM micrographs of the (a,b) side‐view a MOF/Mont extrudate at different magnifications, as well as an EDX map of micrograph (c,d) showing Si (blue), Al (pink) and C (yellow) dispersed throughout the material within the cracks observed. Micrographs of the indentations created by the Ga^+^ beam on the longitudinal cross‐section at different magnifications (e) and (f), revealing the pores probed by Hg porosimetry. The triangles in the inset schemes indicate the position that was imaged by the electron beam.

On the other hand, in the case of the montmorillonite clay, the extrudates showed the presence of cracks on the cylinder (Figure 5a,b), although it is unclear at this point if they arise during the extrusion, or the calcination process. Figure 5b,c shows a rougher surface composed of nanosized crystallites and larger stepped aggregates. Elemental mapping analysis was carried out to reveal the location of MOF and binder material. Elemental X‐ray dispersive spectroscopy (EDX) mapping Figure 5d shows the presence of Si (blue, binder); Al (pink, EDX) and C (yellow, MOF) domains of several microns. Moreover, the packing of MOF and montmorillonite appear less effective compared to the bentonite binder. Those observations are consistent with the crush strength tests, which showed MOF/Mont extrudates are less robust than MOF/Bent ones. Further imaging of the longitudinal cross‐section (Figure 5e,f) allows for the visualization of the larger mesopores in the 10–100 nm range, evidenced by Hg porosimetry and thus corroborating their size and morphology.

In summary, the MOF/clay extrudates show high mechanical stability and effective packing. However, this occurs at the expense of porosity. The higher porosity is observed when silica gel is used as binder and it can be further enhanced using a longer calcination period (18 h). However, the use of this binder is detrimental to the mechanical stability that remain poor whatever the calcination time.

### Density and CO_2_ and CH_4_ Adsorption Isotherms

**Figure 6:**
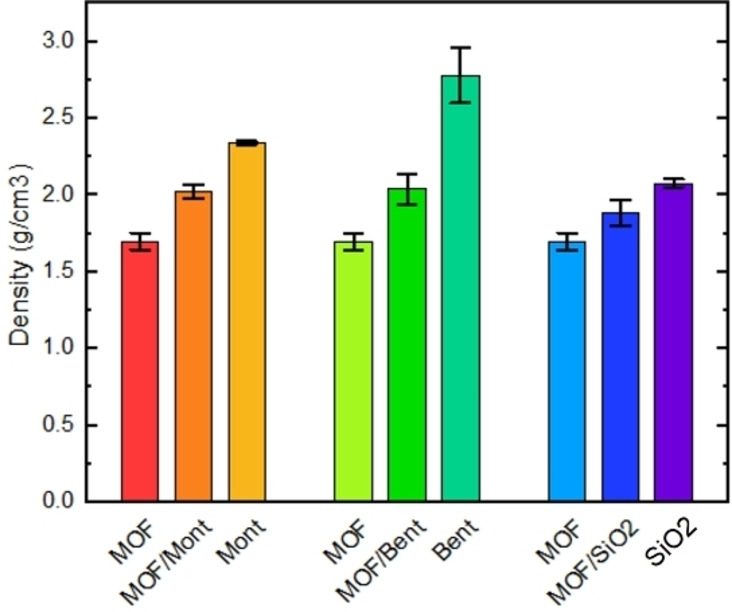
Skeletal density values of the MOF, MOF/binder and pure binder extrudates determined by He pycnometry at 313 K.

The extent of powder compaction was evaluated by means of He pycnometry as shown in Figure 6. If one assumes a zero excess‐volume of mixing, it is possible to recalculate the composition of the extrudates. This leads to 49/51, 68/32 and 51/49 of the MOF/Mont, MOF/Bent and MOF/SiO_2_ extrudates, respectively. This indicates that in the case of bentonite, a higher skeletal compaction of the clay‐MOF mixture is achieved with respect to the other binders, which can be potentially ascribed to the easier mixing and lower viscosity during extrusion.

**Figure 7:**
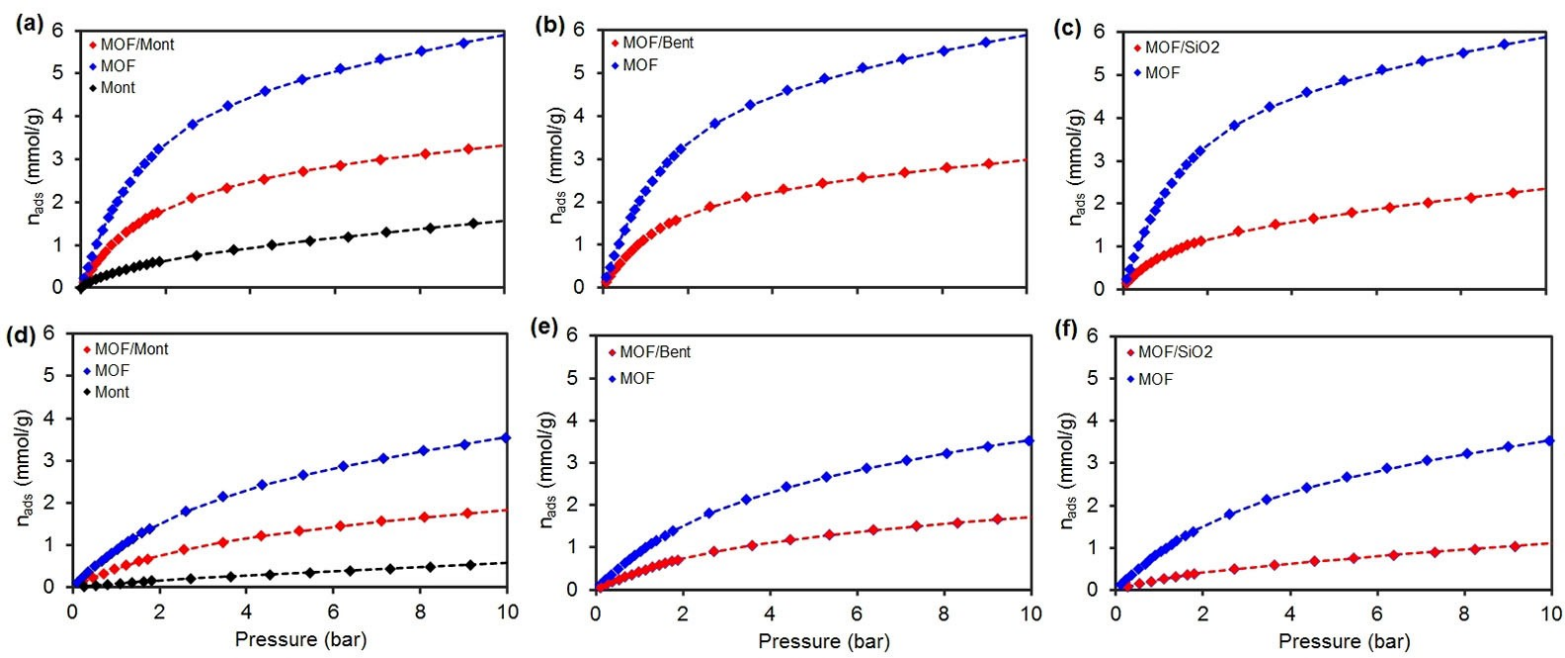
Single‐component adsorption isotherms of (a–c) CO_2_ and (d–f) CH_4_ at 303 K with the experimental data (filled diamonds, **�**) in the p=1–10 bar range and the fitted curves to a 3‐site Jensen‐Seaton (doted lines, ‐ ‐ ‐ ) model isotherm.

The most promising application that has been studied in the case of aluminum fumarate is water sorption for heat exchangers.[^23^, ^24^] Other than heat pumps, a number of patents on the adsorption properties of CO_2_, CH_4_ and H_2_ with aluminum fumarate have been reported.[^12^, ^44^] To evaluate the potential of the extrudates as sorbents, CO_2_ and CH_4_ adsorption isotherms at 303 K were measured up to 10 bar (Figure 7). The adsorption capacity for CO_2_ is higher than that for CH_4_ in all cases, as previously explained by theoretical calculations and experiments.^[46]^ The smaller kinetic diameter of CO_2_ and the higher adsorption enthalpy (−24 vs. −15 kJ ⋅ mol^−1^, respectively), as well as the liquefaction enthalpies (−17.2 vs. −8.2 kJ ⋅ mol^−1^, respectively) lead to more effective surface‐adsorbate interactions. Dispersive forces between CO_2_ and the oxygen and hydroxyl groups explain this; although the presence of Al^3+^ coordinatively unsaturated sites (CUS) due to defects may not be excluded.[^26^, ^36^, ^47^] Porous materials used for separation processes typically combine selectivity values higher than 30, and a working capacity higher than 1 mmol ⋅ g^−1^.[^48^, ^49^] As seen in the isotherms in Figure 7 and Table 2, both MOF/binders and MOF powder adsorb CO_2_ and CH_4_ in moderate quantities compared to other adsorbents reported in the literature.[^50^, ^51^] Thanks to the pure component adsorption isotherms a separation factor was roughly calculated as the ratio CO_2_/CH_4_ of the amount of CO_2_ adsorbed divided by the amount of CH_4_ adsorbed at a given pressure (1 and 5 bars) at 303 K. From the single gas adsorption isotherms, the selectivity of CO_2_ over CH_4_ could be predicted with a thermodynamic model, i. e. Ideal Adsorbed Solution Theory (IAST), in the range of pressure 1–10 bars. Indeed, adsorption selectivities for equimolar CO_2_/CH_4_ mixtures were predicted using the pure component isotherm fits,^[52]^ defined by (Eq. 1):

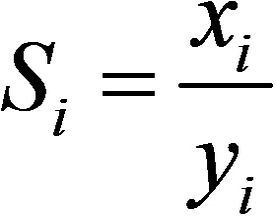




**Table 2:** IAST selectivity, molar adsorption capacities and separation factors obtained from the isotherms of pure CO_2_ and CH_4_ at 303 K (simulation of landfill gas separation via PSA) for the MOF powder, MOF/clay and MOF/SiO_2_ extrudates.

Sample	Selectivity (Jensen‐Seaton)		n_*ads*_, CO_2_ [mmol ⋅ g^−1^]		n_*ads*_, CH_4_ [mmol ⋅ g^−1^]		Separation Factor CO_2_/CH_4_	
Pressure (bar)	1	5	1	5	1	5	1	5
MOF/Mont	3.92	4.39	1.29	2.70	0.31	1.32	4.16	2.04
MOF/SiO_2_	5.39	5.51	0.80	1.72	0.19	0.71	4.21	2.42
MOF/Bent	3.52	4.02	1.11	2.42	0.41	1.28	2.71	1.89
MOF	3.12	3.67	2.25	4.86	0.76	2.60	2.96	1.87

in which *S*_*i/j*_ is the selectivity towards the component *i* in binary mixture with *j* (*i* stands for CO_2_ and *j* for CH_4_), $x_{i}$ and $y_{i}$ denote the equilibrium mole fraction for component *i* in the adsorbed and the gas phase, respectively. These conditions are typically used for landfill gas separation in pressure swing adsorption (PSA) processes. Adsorption of CH_4_ in pure bentonite extrudates was too low to carry out the computation (which would lead to an extremely high separation factor). For the rest of the materials, the experimental curves were fitted using 3‐sites (i. e. MOF, defects, binders) Jensen‐Seaton model isotherms which provided low squared sum of errors at low pressures. All the details of the fitting procedure are given in Supporting Information, section 8 and Table S1.^[53]^ The amount of moles as a function of pressure is given by Equation 2:

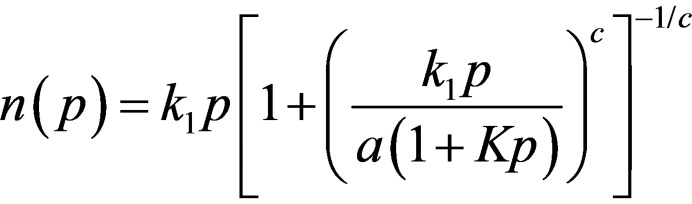




in which *p* is the pressure, *k_1_* is the Henry constant of the solid, *K* is the compressibility of the gas and *c* is a positive empirical constant. As expected, the gas uptake per gram of material was lower for the shaped MOFs than for the powdered sample, since it has often been reported that binders cause pore blocking. It is worth mentioning that this higher capacity depends on the bulk density (i. e. volumetric instead of gravimetric capacity), which varies depending on the components of the shaped adsorbent.^[52]^ Nevertheless, some adsorption properties, such as the CO_2_ selectivity towards CH_4,_ are maintained with the shaping. Even if these estimated values are low, they are very similar to those obtained for the powder MOF.

### Effects on the Acidity of the Adsorbents

As already mentioned, the presence of Lewis or Brønsted acid sites inside the MOFs’ pores can play a dominant role in the adsorption process of gases. The bridging *μ*‐OH are mild acid groups tiled along the lozenge‐like 1D channels able to catalyze, for instance, ethanol dehydration.^[36]^ The impact of binders and extrusion on the nature and the amount of acid sites in microporous crystalline aluminosilicates has been well documented.[^34^, ^54^]

**Figure 8:**
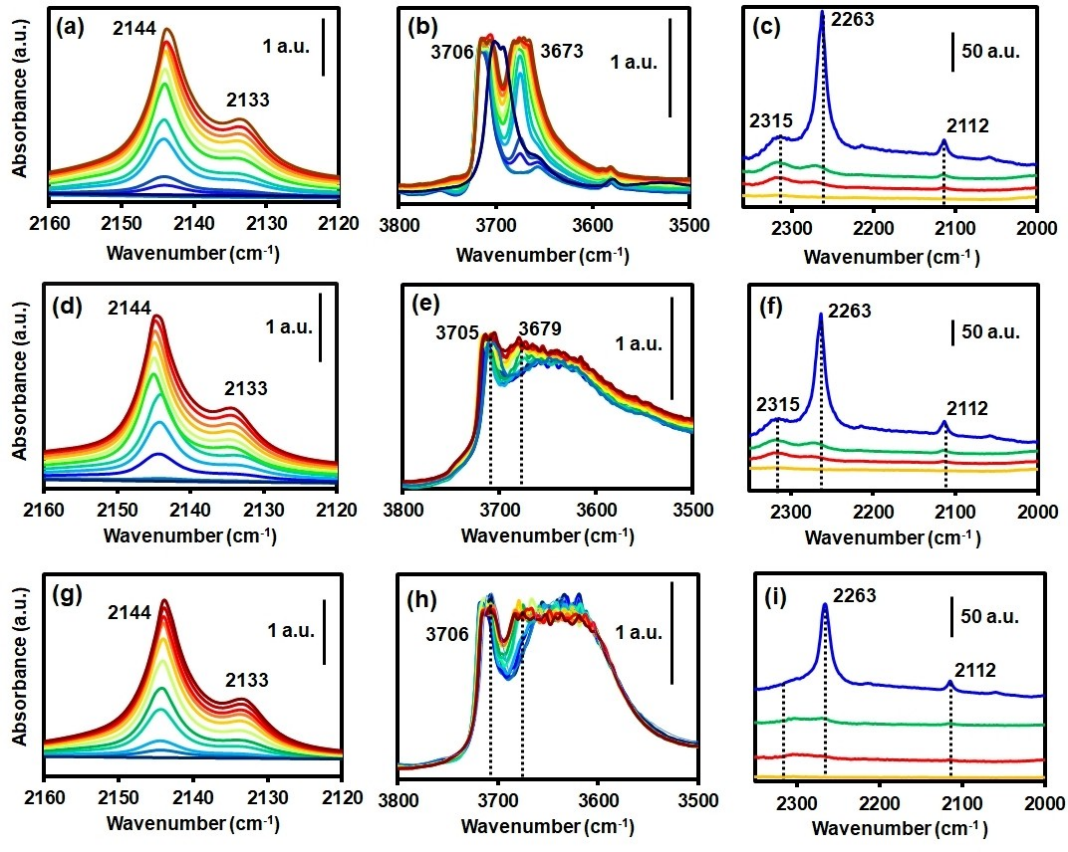
Different regions of the FTIR spectra with increasing CO pressures (dark blue to brown) at 85 K of (a, b) the pure MOF, (d, e) MOF/Mont and (g, h) MOF/Bent extrudate materials. FTIR spectra of (c) the MOF, (f) MOF/Mont and (i) MOF/Bent extrudates after saturation with CD_3_CN (blue), vacuum evacuation at 298 (green), 323 (red) and 423 K (yellow).

Further, montmorillonite consists of magnesium‐aluminum silicates with the general composition (Na,Ca)_0.33_(Al,Mg)_2_Si_4_O_10_(OH)_2_⋅*n*H_2_O; where cations can be replaced by H^+^ by acid treatment.^[55]^ Thus, to understand how extrusion may affect acid sites in the extrudates probed FTIR spectroscopy measurements were performed. Figure 8 shows the FTIR spectroscopy after dosing CO at 85 K and CD_3_CN desorption as probe molecules that allows for the determination of the strength of Brønsted or Lewis sites, respectively.[^56^, ^57^, ^58^] Figure 8a,b show the regions of the main CO and the OH vibrations, respectively, in the spectra of aluminum fumarate upon insertion of CO. In the former, the bands correspond to physisorbed CO (2144 cm^−1^) and OH⋅⋅⋅CO adducts (2133 cm^−1^); which correlate with a split of the main OH vibration at 3690 cm^−1^ into two bands at 3706 and 3673 cm^−1^. From these values (Δν_CO_=11; Δν_OH_=33 cm^−1^), and as previously reported in the literature, it can be concluded that the OH groups are of low strength. Moreover, saturation of the material with CD_3_CN resulted in the appearance of the mode at 2112 cm^−1^, corresponding to the ν_s_(CD_3_) of both *d_3_*‐acetonitrile; and the main ν_s_(C≡N) mode at 2263 cm^−1^.

**Figure 9:**
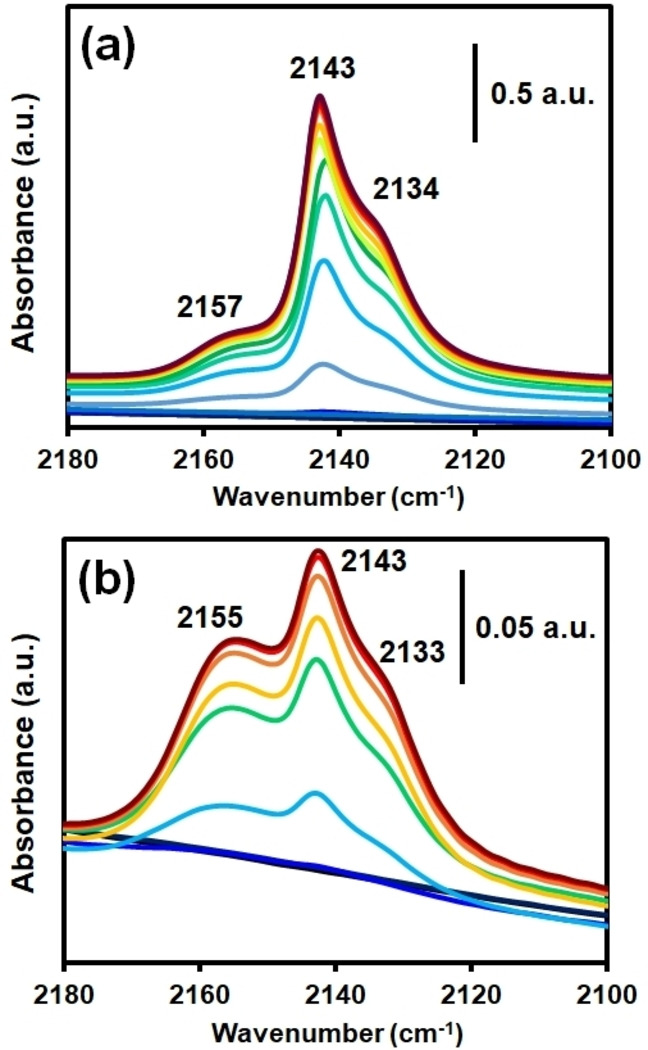
CO‐probe FTIR spectra at 85 K and increasing pressures of the MOF/SiO_2_ extrudates after calcination at 573 K for (a) 5 h and (b) 18 h in air flow.

A band at 2315 cm^−1^ appears as well, assigned to the presence of polarizing, hard Lewis Al^3+^ acid sites, either from undercoordinated lattice sites, extra‐framework cations or nano‐sized *γ*‐Al_2_O_3_.^[59]^ They correspond to relatively strong sites as suggested by the necessity of temperatures up to 323 K and secondary vacuum to completely desorb CD_3_CN from them. These sites are already present in the pure MOF, indicating the presence of defects in the commercial material, probably related to the industrial synthesis process.^[37]^ Identical sites can be observed by both techniques in the case of MOF/Mont extrudates, as seen in Figure 8d–f. In the case of the OH region spectra, the well‐defined peak corresponding to the bridging *μ_2_*‐OH groups overlaps with interlayer hydronium and H_2_O molecules of the clay structure, although indeed a small feature corresponding to peak splitting can be observed. Moreover, Figure 8i shows no presence of the mentioned band at 2315 cm^−1^, indicating the absence of the Al^3+^ Lewis sites. This may be related to the higher tendency of bentonite towards cation exchange of its interlayer species. In the case of silica gel (Figure 9), an additional peak at 2157 cm^−1^, corresponding to silanol surface groups Si−OH⋅⋅⋅CO,^[60]^ was observed, together with those at 2143 and 2133 cm^−1^ previously discussed. This is in line with the PXRD data (shown before in Figure 2) that shows an important decrease in crystallinity. Thus, although the porosity of the material is enhanced, the acidity is strongly decreased by the longer calcination treatment. It can be concluded that the extrusion process with silica gel does not affect the acid sites significantly (as seen for the clays), but longer calcination treatments have a negative impact by partially collapsing the MOF structure, thus, decreasing the amount of bridging *μ*‐OH groups. In the case of pyridine as a probe molecule, little information was obtained, as most of the fingerprint bands appear in the region where either MOF or clays show strong absorbance.

Nevertheless, it helped us to corroborate the weak acidity of the bridging *μ*‐OH groups, as a broad band corresponding to weak Py⋅⋅⋅H^+^ bonding appeared in the 3000–3500 cm^−1^ region (see Figure S9). In the case of montmorillonite, pyridine‐probe FTIR (Figure S9b) showed the typical band at 1637 cm^−1^ corresponding to Brønsted sites, as well as those at 1624, 1490 and 1445, indicating the presence of Lewis sites.^[61]^ These bands were also present in the case of bentonite, proving that both binders have a myriad of cations on the surface in the form of acid sites. However, these are not expected to be in large quantities given the low adsorption capacities observed in the isotherms. It is worth mentioning that we attempted to use NH_3_‐TPD to quantify the amount of acid sites in the MOF‐containing materials (Figure S10). Nevertheless, the combination of poor NH_3_ adsorption and low decomposition temperature limited the applicability for this purpose, contrary to what has been previously reported.^[62]^

## Conclusions

It has been shown that millimeter‐sized bodies containing up to 50 wt % of aluminum fumarate MOF adsorbent can be prepared by an extrusion technique and that clay or SiO_2_ gel can be used as binders. XRD of the ground extrudates show that the crystal structure and porosity are mostly retained, with low evidence of pore blockage. However, SiO_2_ offers poor mechanical properties with respect to the clay materials, which conferred high axial crushing strength.

SEM imaging revealed that bentonite is compacted more tightly than montmorillonite with the MOF particles, which was also corroborated by means of He pycnometry. Adsorption isotherms of CO_2_ and CH_4_ at 303 K showed a promising performance in terms of capacity and selectivity. Indeed, even if the estimated values are still low for application, they are very close to those obtained for the powdered MOF. Moreover, CO_2_ selectivity towards CH_4_ is maintained with the shaping which is an important result. At last, probe FTIR spectroscopy shed light on the effects of extrusion on acidity, which seemed mostly unaffected in the case of clay binders. SiO_2_ gel showed the presence of additional acid sites with longer calcination times, pointing to the generation of additional Lewis sites.

This work, thus, demonstrates that clays could well be used for producing robust extrudates without critically compromising the physicochemical properties of the MOF material that may undermine their use as sorbents. This work highlights the need of carrying out comprehensive characterization to ensure that materials remained unchanged upon scale‐up and paves the way for further work on shaping powders as technical bodies.

## Experimental Section

**Preparation of the MOF extrudates**: The powder pastes with adjusted viscosities were prepared by adding dropwise DI water (18.2 MΩ⋅cm at 298 K, Metrohm Autolab) into a mixer (Caleva Mixer Torque Rheometer 3). The pastes were then transferred into a Mini‐Screw Caleva Multi Lab extruder and extruded through a 2 mm diameter (Ø) die with a screw rotation speed of 90–120 rpm. **MOF/clay extrudates**: For those synthesis, aluminium fumarate (5.0 g, MOF Technologies®, Belfast, UK), either bentonite (5.0 g, SWy‐3, Source Clays Repository, The Clay Minerals Society) or montmorillonite clay (5.0 g, K‐10, Sigma‐Aldrich) and deionized (DI) water (6.0 mL) were put into the mixer, then extruded into the shaped bodies following the procedure above. Reference extrudates of the pure bentonite and montmorillonite were prepared by mixing the clay (10.0 g) with DI water (10.0 g). **MOF/SiO_2_ extrudates**: In this case, aluminium fumarate (5.0 g), silica gel (5.0 g, Grace & Co., Davisil® 1302 Grade), methylcellulose (1.0 g, Sigma‐Aldrich, η=4000 cP) and 1‐amino‐2‐propanol (13.5 mL, 9 wt % aq. Solution, Sigma‐Aldrich, 93 %) were mixed and extruded.

**Characterization and analytical methods**: **powder X‐ray diffraction (XRD)** patterns were obtained using a Bruker‐AXS D2 Phaser powder X‐ray diffractometer in Bragg‐Brentano geometry, using Co K_*α1,2*_=1.79026 Å and operating at 30 kV. Acquisitions were carried out between 5 and 70 using a step size of 0.05° and an acquisition time of 1 s per point. **N_2_ adsorption isotherms** at 77 K were measured with a Tristar 2020 instrument from Micromeritics. Prior to the measurement, the samples were pre‐treated in a 1 mL ⋅ min^−1^ stream of N_2_ at 573 K for 10 h to remove any adsorbed water or other impurities. **Hg porosimetry intrusion** measurements were carried out using an AutoPore IV 9500V1.09 instrument (Micromeritics®). Prior to the intrusion cycles, the extrudates were degassed in N_2_ flow at 353 K overnight, followed by 15 min under secondary vacuum. Surface tension (*γ*) and contact angle (θ) were set to 485 dyn ⋅ cm^−1^ and 140°, respectively, and the materials infiltrated up to P_max_=60 000 psi to probe pores of a minimal diameter Ø=4 nm. The samples were submitted to one intrusion‐extrusion cycle, and Washburn equation $p=-\frac{4\gamma cos\theta }{d}$ was applied in the pressure range probed.

**Focused ion beam scanning electron microscopy coupled with energy‐dispersive X‐ray spectroscopy (FIB‐SEM‐EDX)** imaging was carried out on a FEI Helios Nanolab 600 DualBeam with an Oxford Instruments Silicon Drift Detector X‐Max energy‐dispersive spectroscope. EDX mapping was performed with an electron beam of 5–10 kV and 0.1–0.8 nA. FIB cuts for cross sectional images were made by covering an area of 5×2 mm^2^ of the substrate with 500 nm of Pt by sputtering at 30 kV and 0.08 nA. Then a cut of 7×5 mm^2^ and 5 mm deep was made with the ion beam at 30 kV and 2.5 nA. **Uniaxial compression crush strength tests** were performed over the lateral surfaces of the extrudate cylinders using a Chatillon Model MT Tension / Compression Mechanical Test instrument in air working at 298 K. **CO‐probe Fourier‐Transformed Infrared (FTIR) spectroscopy** measurements were recorded on a PerkinElmer System 2000 instrument (16 scans, 4 cm^−1^ resolution, DTGS detector, cell with KBr windows). Typically, ∼20 mg of sample was pressed into ∼2 cm^2^ pellets in a press‐tool, then activated under secondary vacuum (p <10^−5^ mbar) at 573 K for 3 h. After cooling down the cell to 85 K with liquid N_2_, a 10 % CO/He v/v (Linde AG, 99.9 %) mixture was introduced into the cell stepwise, the spectra recorded at the following pressures: 0.065, 0.1, 0.45, 0.84, 4.8, 10, 40, 50, 70, 75, 100 and 150 mbar. **Py‐probe FTIR spectroscopy** experiments of MOF‐including materials were carried out in a ThermoFischer Nicolet *i5* spectrometer (32 scans, 4 cm^−1^ resolution, DTGS detector, cell with KBr windows) on the same pellets used for CO experiments. After activation under secondary vacuum (p<10^−5^ mbar) at 573 K for 3 h, pyridine (99 %, re‐distilled, Sigma‐Aldrich) was introduced (10 mbar) into the cell and the spectra recorded at selected times. **CD_3_CN‐probe FTIR spectroscopy** experiments were carried out in a Nicolet 6700 spectrometer (16 scans, 4 cm^−1^ resolution, DTGS detector, cell with KBr windows). The pellet samples were pre‐treated in the same manner as for CO and Py probed spectroscopy. Then, the cell was saturated with CD_3_CN (+99.8, Sigma‐Aldrich) for 1 h. Thereafter, the pellets were evacuated with vacuum, then with increasing temperatures up to 423 K and the spectra recorded at the described temperatures. **Gas adsorption measurements** at 303 K and pressures up to 10 bars were made with CO_2_ and CH_4_ (quality N48 and N55, respectively) using a homemade high‐throughput instrument.^[63]^ In this set‐up, gas dosing is made on six samples in parallel with a manometric gas dosing system. The samples were activated at 443 K for 16 h individually in situ under primary vacuum overnight. Around 100 mg of sample were used for each extrudate composition. Helium (N55) was used to measure the dead volume inside the cells. **Density measurements** were carried out by means of helium pycnometry. The experiments were performed at 313 K on a BELSORP Max 1 apparatus. For one measurement, the final value is the average value over ten cycles. For each sample, three measurements were performed.

## Conflict of interest

The authors declare no conflict of interest.

## Supporting information

As a service to our authors and readers, this journal provides supporting information supplied by the authors. Such materials are peer reviewed and may be re‐organized for online delivery, but are not copy‐edited or typeset. Technical support issues arising from supporting information (other than missing files) should be addressed to the authors.

Supporting Information
