# Supplementary material for: Understanding the Effects of Binders in Gas Sorption and Acidity of Aluminium Fumarate Extrudates
Source: Chemistry. 2021 Dec 10;28(5):e202103420. doi: 10.1002/chem.202103420 (PMC9299853; doi:10.1002/chem.202103420)
Supplement: Supplementary file 1 — Supporting Information [file CHEM-28-0-s001.pdf]

Chemistry–A European Journal

Supporting Information

Understanding the Effects of Binders in Gas Sorption and Acidity of Aluminium Fumarate Extrudates

Miguel Rivera-Torrente, Danny Kroon, Marie-Vanessa Coulet, Carlos Marquez, Nikolaos Nikolopoulos, Rifan Hardian, Sandrine Bourrelly, Dirk De Vos, Gareth T. Whiting,* and Bert M. Weckhuysen*

SUPPORTING INFORMATION

Table of Contents

1. **Materials and Methods**
2. **X-ray Diffraction (XRD) of pure Aluminum Fumarate Powder**
3. **N_2 Adsorption Isotherms at 77 K of the pure Aluminum Fumarate Powder**
4. **Thermogravimetric Analysis (TGA) of the pure Aluminum Fumarate Powder**
5. **X-ray Diffraction (XRD) of MOF/SiO_2 Extrudates after Calcination**
6. **Hg Intrusion of the Pure Montmorillonite and Bentonite Binders**
7. **N_2 adsorption isotherms at 77 K of the SiO_2 Gel Powder**
8. **Fits to Jensen-Seaton Model Adsorption Isotherms of CO_2 and CH_4 at 303 K**
9. **FT-IR Spectroscopy of Pure Aluminum Fumarate, Montmorillonite and Bentonite Powders**
10. **NH_3 Temperature Programmed Desorption of the MOF Powder and MOF/Mont Extrudates**

1. Materials and Methods

Materials and methods: aluminum fumarate [$Al(C_4H_4O_4)(OH)_2 \cdot nH_2O$] was purchased from MOF Technologies™ (Belfast, UK) and dried at 473 K under dynamic vacuum (p < 0.1 bar) for 24 h prior to use. Montmorillonite (aluminum-pillared clay, Sigma-Aldrich, 250 m²/g) and bentonite (SWy-3, Source Clays Repository, The Clay Minerals Society) were obtained from commercial sources and used without further purification. Composition of the mineral bentonite clay was studied by inductively coupled plasma-optical emission spectrometry (ICP-OES) analysis, showing an elemental composition (%) of Al, 10.6; Fe, 2.78; K, 0.45; Mg, 1.44; Na, 1.4; P, 0.02 and Si, n.a. Silica gel (SiO_2, Grace & Co., Davisil® 1302 Grade) and 1-amino-2-propanol (Sigma-Aldrich, 93%). Methylcellulose (Sigma-Aldrich, η = 4000 cP).

Thermogravimetric analysis (TGA) experiments were performed on a TGA Q50 V6.7 Build 203 (TA Instruments, USA). The sample was placed under a 100 $mL \cdot min^{-1}$ flow of a 79/21 (v/v%) N_2/O_2 (synthetic air) gas mixture and heated according from 303 to 1273 K at a rate of 15 $K \cdot min^{-1}$.

Inductively Coupled Plasma-Optical Emission Spectroscopy (ICP-OES) has been performed by the Geolab (Utrecht University), using a SPECTRO CIROSCCD (by SPECTRO Analytical Instruments GmbH–Germany). Clays were dissolved using an aqua regia with HF (Sigma-Aldrich, aq. 48-52% v/v) solution, in which they were dissolved at 363 K overnight, after which it was cooled down to 298 K and neutralized using boric acid. After this the solutions were diluted to yield to appropriate concentrations.

Temperature programmed desorption of ammonia (NH_3-TPD): was performed on a Micromeritics Autochem II 2920 equipped with a thermal conductivity detector (TCD). Prior to the TPD experiment, *ca.* 0.1 g of material was outgassed under a He flow for 1 h at 523 K with a heating ramp of 10 $K \cdot min^{-1}$, then cooled to 373 K. Ammonia was adsorbed at 373 K until saturation, followed by flushing with He for 120 min at 373 K. The ammonia desorption was monitored using the TCD detector until 823 K with a ramp of 5 $K \cdot min^{-1}$, using a flow of 25 $mL \cdot min^{-1}$.

2. X-ray Diffraction (XRD) of pure Aluminum Fumarate Powder

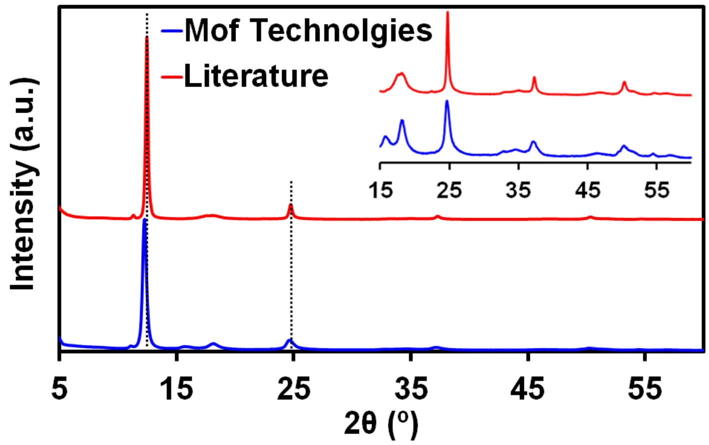

Figure S.1. X-ray diffraction patterns of the commercial aluminum fumarate (MOF Technologies™, blue) and lab-made aluminum fumarate following the microwave-assisted procedure at 403 K reported in reference [1].

3. N_2 Adsorption Isotherms at 77 K of the pure Aluminum Fumarate Powder

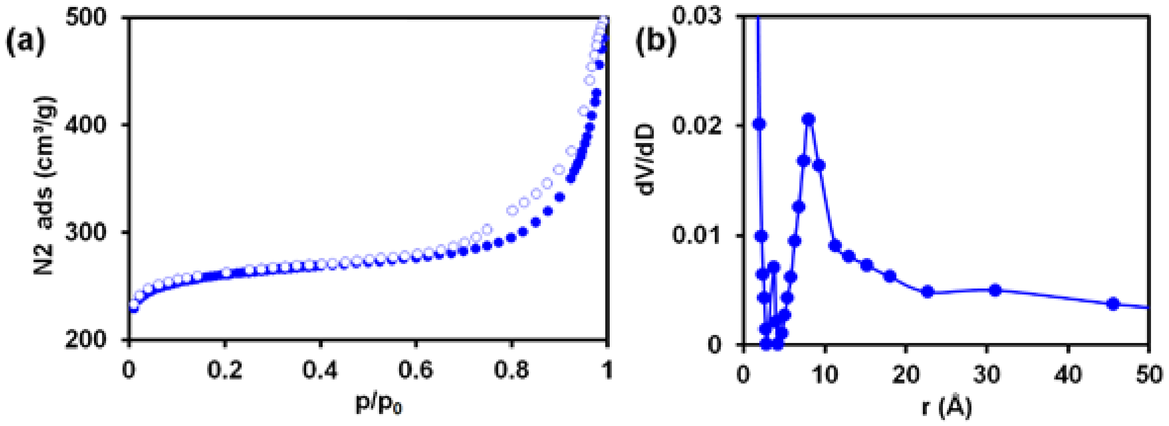

Figure S.2. (a) N_2 adsorption isotherms at 77 K (adsorption, filled dots; and desorption, open dots) and (b) pore size distribution obtained from the BJH method of the pure commercial aluminum fumarate (MOF Technologies^{TM}, blue).

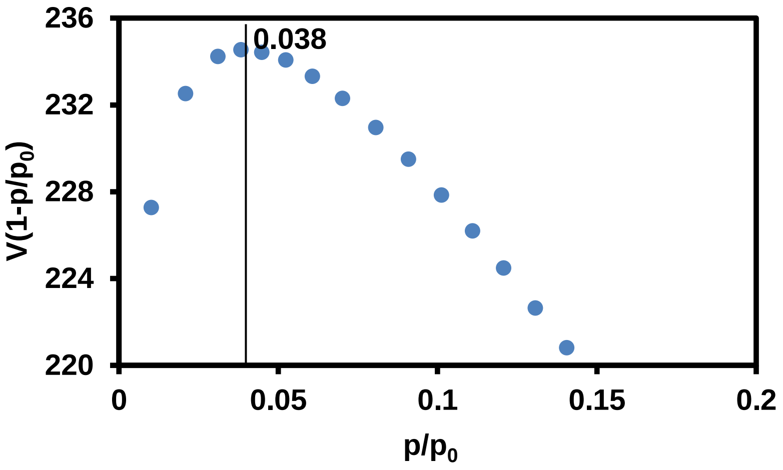

Figure S.3. Roquerol plot showing $V(1\text{-}p/p_0)$ as a function of relative pressure p/p_0 to determine the minimum relative pressure value that may be used for the linear BET fit.

The BET surface areas have been calculated using the Rouquerol criterion.[2] The Rouquerol plot, which is $V(1\text{-}p/p_0)$ as a function of p/p_0, has been plotted to identify the first maximum pressure useful for a linear fit to the BET equation (Eq. 1).

$$\frac{\frac{p}{p_0}}{V(1-p/p_0)} = \frac{1}{V_m \cdot C} + \frac{C-1}{V_m C}(p/p_0) \qquad (1)$$

The adsorption data are then fit to the BET equation (Eq. 1) by a linear regression (y = ax + b). The slope a and the intercept b are used to calculate the monolayer volume (V_m) and then the BET surface area (S_{BET}) with the following equations (Eq. 2,3):

$$V_m = 1/(a+b) \qquad (2)$$

$$S_{BET} = \sigma_{N2} \frac{V_m}{V_{N2}} N_A \qquad (3)$$

$$S_{BET}\ (m^2/g) = 4.36\ V_m\ (mL\ STP) \qquad (4)$$

, where V_{N2} is the molar gas volume, N_A is Avogadro number, and σ_{N2} is the cross-section of the N_2 molecule, which corresponds experimentally to (Eq. 4) for σ_{N2} = 0.162 nm^2 :

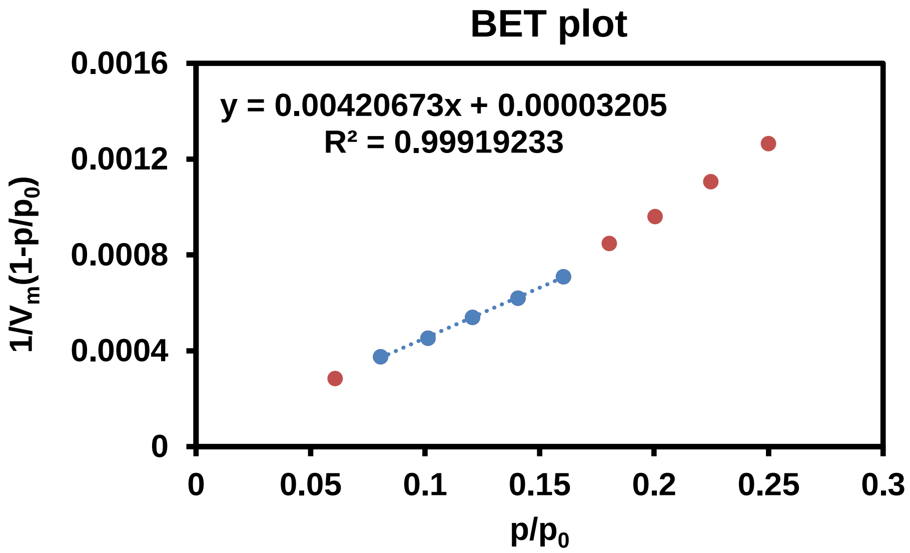

Figure S. 4. BET plot of the linear fit including the selected range used (according to the Rouquerol consistency criteria)[2] for the fit represented by the dots in blue.

4. Thermogravimetric Analysis (TGA) of the pure Aluminum Fumarate Powder

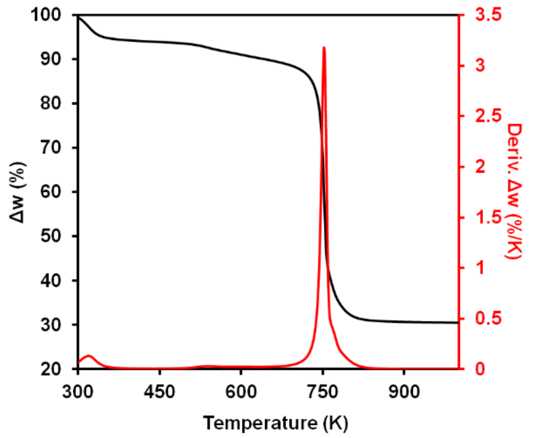

Figure S.5. Thermogravimetric analysis of the pure commercial aluminum fumarate under a 100 mL·min^{-1} flow of a 79/21 (v/v%) N_2/O_2 (synthetic air) gas mixture and heated according from 303 to 1273 K at a rate of 15 K·min^{-1}.

5. X-ray Diffraction (XRD) of MOF/SiO_2 Extrudates after Calcination

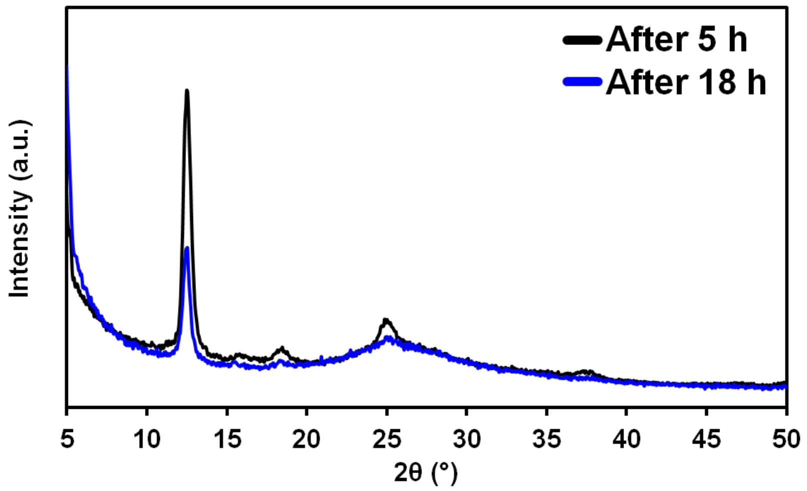

Figure S.6. Powder X-ray difractograms of the MOF/SiO_2 extrudates after calcination at 598 K for 5 h (black) and 18 h (blue).

6. Hg Intrusion of the Pure Montmorillonite and Bentonite Binders

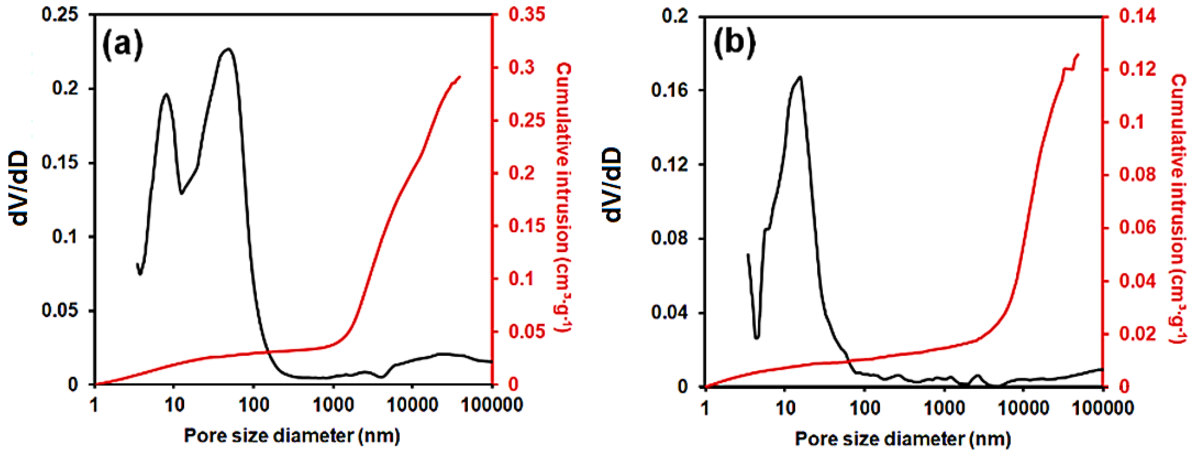

Figure S.7. Hg porosimetry intrusion (red); and pore size distribution (black) curves of the extrudates prepared only clay (a) montmorillonite and (b) bentonite.

7. N_2 adsorption isotherms at 77 K of the SiO_2 Gel Powder

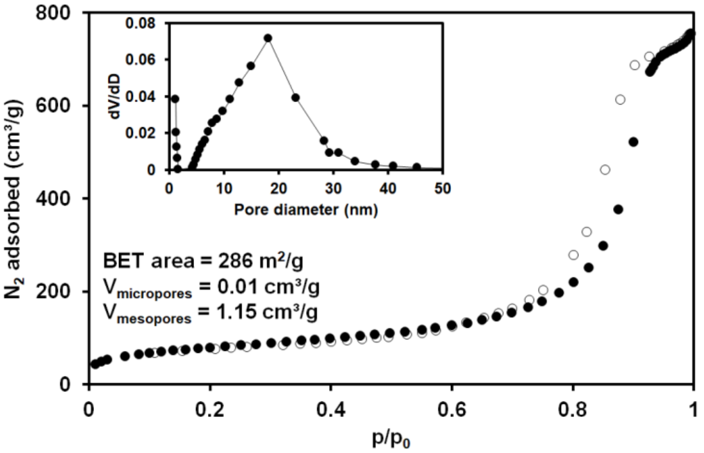

Figure S.8. N_2 adsorption isotherm at 77 K of the pure SiO_2 gel binder (inset shows the pore size distribution calculated by the BJH method).

8. Fits to Jensen-Seaton Model Adsorption Isotherms of CO_2 and CH_4 at 303 K

Table S. 1. Adsorption Isotherm Data of CO_2 and CH_4 at 303 K and Jensen-Seaton Fit with Calculated Parameters and Squared Sum Residual (SSR) of the pure Aluminum Fumarate powder.

Material	Coefficients	CO2 Fit Jensen-Seaton	CH4 Fit Jensen-Seaton
Aluminum Fumarate powder	a	4.90	3.19
	K	2.98	1.03
	c	1.40	1.32
	k	0.03017	0.0376
	**Σ diff**	**0.00096**	**0.0016**
Montmorillonite extrudates	a	2.10	0.07
	K	1.05	0.79
	c	0.55	6.92
	k	0.0630	0.6944
	**Σ diff**	**0.0011**	**0.0204**
MOF/Mont extrudates	a	3.40	1.61
	K	1.96	0.50
	c	0.99	1.33
	k	0.0187	0.0042
	**Σ diff**	**0.0004**	**0.0011**
MOF/Bent extrudates	a	2.54	1.35
	K	1.61	0.47
	c	1.25	1.57
	k	0.0297	0.0485
	**Σ diff**	**0.0017**	**0.0009**
MOF/SiO2 extrudates	a	1.77	0.48
	K	1.56	0.29
	c	0.94	1.82
	k	0.0629	0.1529
	**Σ diff**	**0.0031**	**0.0053**

9. FT-IR Spectroscopy of Pure Aluminum Fumarate, Montmorillonite and Bentonite Powders

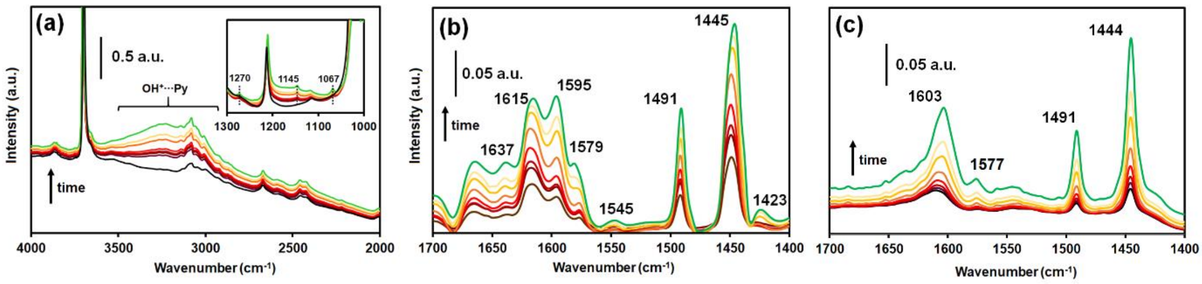

Figure S.9. FT-IR spectra showing observable peaks of (a) the pure aluminum fumarate, (b) montmorillonite and (c) bentonite clays after adsorption of pyridine from 1 min (black) to 120 min (green). Inset in (a) shows fundamental modes of acid-bonded pyridine in the 1300-1000 cm^{-1} region. Baseline correction has been applied in the spectra depicted in (b) and (c).

10. NH_3 Temperature Programmed Desorption of the MOF Powder and MOF/Mont Extrudates

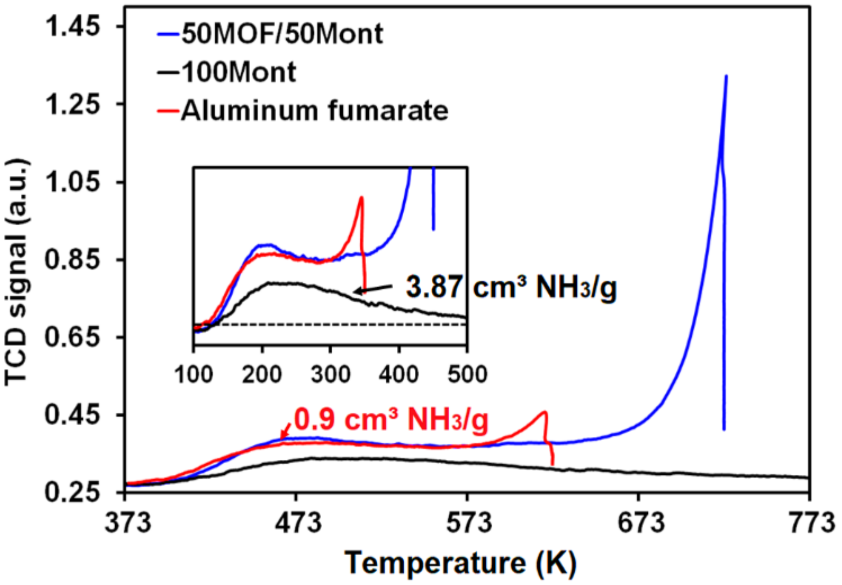

Figure S.10. NH_3-TPD plot of aluminum fumarate and the montmorillonite extrudates showing the amount of acid sites. The sharp peaks beyond 573 K correspond to material decomposition (forming the CO_2 observed in the TCD detector).

11. Notes and References

1. E. Alvarez, N. Guillou, C. Martineau, B. Bueken, B. Van de Voorde, C. Le Guillouzer, P. Fabry, F. Nouar, F. Taulelle, D. de Vos, J.-S. Chang, K. H. Cho, N. Ramsahye, T. Devic, M. Daturi, G. Maurin and C. Serre, *Angew. Chem. Int. Ed.*, 2015, **54**, 3664-3668.
2. J. Rouquerol and F. Rouquerol, in *Adsorption by Powders and Porous Solids (Second Edition)*, Academic Press, Oxford, 2014, pp. 57-104.
